# UGT2B17 and miR-224 contribute to hormone dependency trends in adenocarcinoma and squamous cell carcinoma of esophagus

**DOI:** 10.1042/BSR20190472

**Published:** 2019-07-05

**Authors:** Xiangyao Lian, Ancha Baranova, Jimmy Ngo, Guiping Yu, Hongbao Cao

**Affiliations:** 1 Department of Oncology, The Affiliated Hospital of Chengde Medical University, Chengde 067000, Hebei Province, China; 2 School of Systems Biology, George Mason University (GMU), Fairfax, VA 22030, U.S.A.; 3 Research Centre for Medical Genetics, Moscow 115478, Russia; 4 Department of Cardiothoracic Surgery, The Affiliated Jiangyin Hospital of Southeast University Medical College, Jiangyin 214400, Jiangsu Province, China; 5 Department of Psychiatry, First Hospital/First Clinical Medical College of Shanxi Medical University, Taiyuan 030001, China; 6 Department of Genomics Research, R&D Solutions, Elsevier Inc., Rockville, MD 20852, U.S.A.

**Keywords:** esophageal adenocarcinoma, Functional Pathway analysis, mega-analysis, multiple linear regression analysis, squamous cell carcinoma

## Abstract

Esophageal squamous cell carcinoma (ESCC) and esophageal adenocarcinoma (EA) are the two main subtypes of esophageal cancer. Genetics underpinnings of EA are substantially less understood than that of ESCC. A large-scale relation data analysis was conducted to explore the genes implicated with either EA or ESCC, or both. Each gene linked to ESCC but not EA was further explored in mega-analysis of six independently collected EA RNA expression datasets. A multiple linear regression (MLR) model was built to study the possible influence of sample size, population region, and study date on the gene expression data in EA. Finally, a functional pathway analysis was conducted to identify the possible linkage between EA and the genes identified as novel significant contributors. We have identified 276 genes associated with EA, 1088 with ESCC, with a significant (*P*<5.14e-143) overlap between these two gene groups (*n*=157). Mega-analysis showed that two ESCC-related genes, *UGT2B17* and *MIR224*, were significantly associated with EA (*P*-value <1e-10), with multiple connecting pathways revealed by functional analysis. ESCC and EA share some common pathophysiological pathways. Further study of UGT2B17 and MIR224, which are differentially dysregulated in ESCC and EA tumors, is warranted. Enhanced expression of UGT2B17 and the lack of miR-224 signaling may contribute to the responsiveness of EA to the male sex steroids.

## Introduction

Esophageal cancer, a lethal disease with poor prognosis, histologically occurring in two major forms: esophageal squamous cell carcinoma (ESCC) and esophageal adenocarcinoma (EA). Although ESCC is reported as the predominant subtype [1], the occurrence of EA has increased over the past 20 years [2]. Both ESCC and EA have distinct geographic patterns of incidence [3,4]. Besides the geographic location, the lifestyle factors such as smoking and alcohol increase the risks of ESCC [5], while EA has been linked to effects of long-term persistence of an acid reflux [6]. Tobacco is reported as a risk factor for both types of esophageal cancer [7].

In both ESCC and EA, multiple genetic and epigenetic alterations have been reported as pathophysiologically important, with many of them also identified as biomarkers for early diagnosis, prognosis or the response to the treatment [8–10]. Notably, a variety of genes with aberrant expression patterns or mutations were implicated in both diseases, even though the etiology for their association remained unclear. For example, alterations in levels of TP53 protein are highly prevalent in EA [11] and also are associated with the progression of ESCC [12]; therefore, mRNA and protein encoded by *TP53* gene are suitable as prognostic biomarkers for both types of malignant esophageal cancer [13,14].

Systems biology approaches that involve the knowledge-based algorithms for analyzing integrated data, predominantly molecular pathways, and networks, are becoming a common aid in inferring novel pathophysiological insights from changes in the levels of various biomolecules profiled in a high-throughput fashion [15,16]. In the present study, we attempted to use a knowledge-based approach to extract information on the pathways shared between ESCC and EA. To that end, we mined existing literature to extract relation data between various human genes and pathophysiology of esophageal tumors, and then performed mega-analysis of existing expression datasets, which allowed us to highlight the products of UGT2B17 and MIR224 genes as involved in the development of EA.

## Materials and methods

This manuscript is organized as follows. First, a large-scale literature-based ESCC-gene and EA-gene relations data were studied, through which EA related genes and ESCC related genes were identified and compared. Then a mega-analysis was conducted for each of these genes that were specifically implicated with ESCC, using 6 out of 109 EA array-expression datasets from Gene Expression Omnibus (GEO). For these genes that showed a significant change in the mega-analysis, a functional pathway analysis was conducted to study their pathogenic significance to EA.

### Literature-based relation data

Literature-based genetic relation data for both EA and ESCC were analyzed. The data were acquired using Pathway Studio (www.pathwaystudio.com) and organized into a genetic database named as **EA_ESCC**, which is available at ‘Bioinformatics Database’ (http://database.gousinfo.com). The downloadable format of the database in Excel is available at http://gousinfo.com/database/Data_Genetic/EA_ESCC.xlsx, which is also available as **Supplementary Material S1** (EA_ESCC.xlsx). Besides the full lists of genes (**EA_ESCC→ EA_specific genes, EA_ESCC→ ESCC_specific genes**, and **EA_ESCC→Common genes**), we also presented the information of supporting references for each disease–gene relation (**EA_ESCC→Ref for EA_specific genes, EA_ESCC → Ref for ESCC_specific genes**, and **EA_ESCC → Ref for Common genes**), including titles of the references and the related sentences where the disease–gene relationship were identified. The information could be used to locate a detailed description of how a candidate gene is associated with EA and/or ESCC.

### Data selection for mega-analysis

The EA array-expression datasets were acquired from GEO (https://www.ncbi.nlm.nih.gov/geo/). After the initial search with keyword ‘esophageal adenocarcinoma’, we identified 109 array-based expression profiling experiments. Then the following criteria were applied to fulfill the purpose of the present study, including (i) the data organism is *Homo sapiens*; (ii) the data type is RNA expression by array; (iii) the study design is EA case vs. healthy control; (iv) the sample size is no less than 10; (v) the dataset and corresponding format files were feasibly available. There were six datasets satisfying the selection criteria and were included for the mega-analysis, as shown in Table 1.

**Table 1:** Datasets used for gene–EA relation mega-analysis

Study name	#Control	#Case	#Sample	Country	GEO ID
Kimchi et al., 2004	GSE1420	8	8	U.S.A.	15
Kim et al., 2011	GSE13898	75	28	U.S.A.	8
Saadi et al., 2010	GSE19529	5	5	United Kingdom	9
Nancarrow et al., 2011	GSE28302	9	23	Australia	8
Ferrer-Torres et al., 2016	GSE74553	13	52	U.S.A.	3
El-Rifai et al., 2016	GSE92396	10	12	U.S.A.	3

### Mega-analysis models

For a gene, the log2 fold change (LFC) of its expression level was used as effect size. Both the fixed-effect model and random-effects model were employed to investigate and compare the effect size [17]. The heterogeneity analysis was conducted to study the variance within and between different studies. In the case that the total variance Q is equal to or smaller than the expected between-study variance df, the statistic ISq = 100% × (Q − df)/Q will be set as 0, and a fixed-effect model was selected for the mega-analysis. Otherwise, a random-effects model was selected. The Q-p represents the probability that the total variance is coming from within-study only.

Significant genes from this mega-analysis were reported, which were identified with the criteria as follows: *P*<1e-7 and effect size (LFC) >1 or < −1. When a gene presents an effect size LFC>1 or < −1 in the mega-analysis, it means that the change of the expression level of the gene is greater than two-fold or smaller than 1/2-fold. While we present all the mega-analysis results in the EA-ESCC**→**mega-analysis, the discussion will be focused on these genes with abs (LFC) > 1. All analysis was conducted by an individually developed MATLAB (R2017a) mega-analysis package. The additional detailed results are online available at (http://gousinfo.com/database/Data_Genetic/EA_ESCC.xlsx).

### Multiple linear regression analysis

An multiple linear regression (MLR) model was employed to study the possible influence of three factors on the gene expression change in EA: sample size, population region and study date. *P*-values and 95% confidence interval (CI) were reported for each of the factors. The analysis was done in Matlab (R2017a) with the ‘regress’ statistical analysis package.

### Functional pathway analysis

For the possible common risk genes identified through expression mega-analysis described above, a functional pathway analysis was conducted between the target genes and both diseases to identify potential biological connections. The analysis was performed using the ‘Shortest Path’ module of Pathway Studio (www.pathwaystudio.com).

## Results

### Genes commonly involved in EA and ESCC

**Figure 1:**
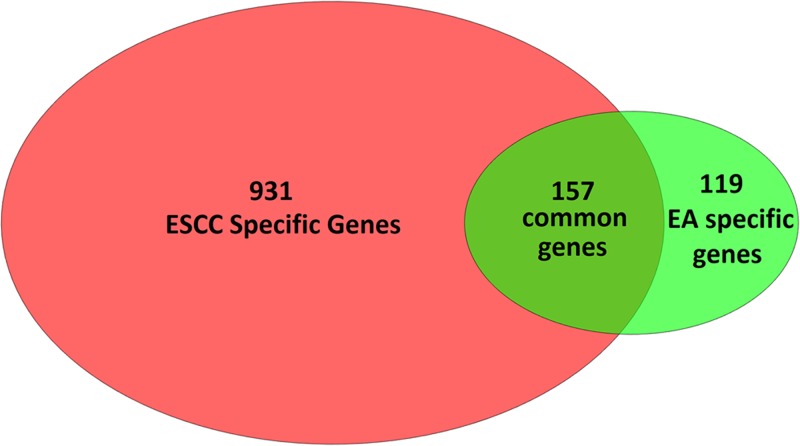
Venn diagram of the gene sets implicated in EA or ESCC in the mined literature

Pathway Studio guided literature mining for the genes associated with EA yielded 276 genes, while ESCC was associated with 1088 genes. These findings were supported by 1335 and 6764 references, respectively. The full list of these genes and the related references can be found in **EA_ESCC** (http://gousinfo.com/database/Data_Genetic/EA_ESCC.xlsx). As shown in Figure 1, a significant overlap (Right tail Fisher’s Exact test, *P*-value = 5.14e-143) of 157 genes was identified for gene sets associated with EA and ESCC, which included more than half of the EA-associated genes (56.88%). Detailed descriptions of these 157 genes are presented in **EA_ESCC→Common genes** and **EA_ESCC→Ref for common genes.**

To explore the functional characteristics of the 157 genes commonly involved in the pathogenesis of both EA and ESCC, we conducted a Gene Set Enrichment Analysis (GSEA) using Pathway Studio, with the 157 genes run as input against the Gene Ontology (GO) and Pathway Studio Ontology. Enrichments were detected in two pathways/gene sets related to apoptosis (78 unique genes), five related to cell growth/ proliferation (86 unique genes), four to protein phosphorylation (67 unique genes), one to transcription factors (57 unique genes), and one to aging (38 unique genes). A complete list of 102 pathways/gene sets enriched with 138 unique genes (FDR correction *P*-value <1e-26) is available at in **EA_ESCC→Common Pathways**.

### Gene expression analysis

**Figure 2:**
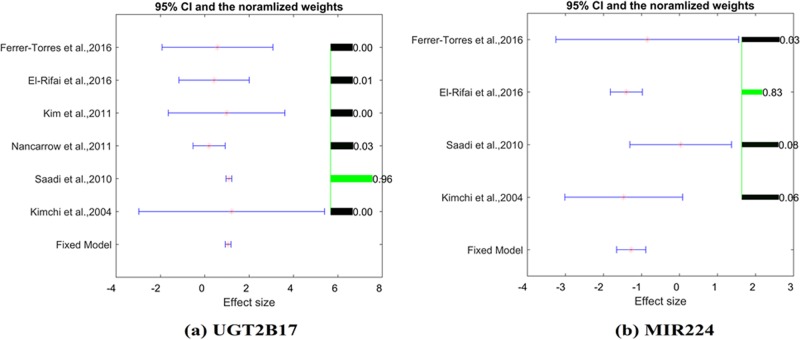
The effect size, 95% CI and weights for genes: UGT2B17 and MIR224 The results are from UGT2B17 and MIR224 mega-analysis performed according to the fixed-effect model.

Despite a significant overlap between EA-genes and ESCC-genes (157 genes; *P*-value = 5.14e-143), a majority of the ESCC-related genes (931 genes, 85.48%) have not been implicated in EA. For each of these 931 genes and EA, the correlations of expression level and the presence of the disease has been tested using six gene expression datasets (see Table 1). The detailed results are presented in **EA_ESCC→ Mega-analysis**. Two genes have passed the significant criteria (*P*<1e-7 and abs (LFC) > 1). For these two genes, a summary of mega-analysis and MLR-analysis could be found in Table 2 and Figure 2, with effect size, 95% CI and weights of each dataset.

**Table 2:** Mega-analysis implicates UGT2B17 and MIR224 genes in pathophysiology of EA

Gene name	Mega-analysis results					MLR analysis		
	Fixed-effect model	Datasets included	LFC	STD of LFC	*P*-value	Sample size	Population region	Study age
*UGT2B17*	Yes	6	1.06	0.07	<1.00e-320	8.13e-3	5.53e-4	5.31e-4
*MIR224*	Yes	4	−1.27	0.20	4.30E-11	<1e-324	<1e-324	<1e-324

To note, the LFC of the genes were estimated from the majority of the selected datasets (six and four studies for *UGT2B17* and *MIR224*, respectively). As shown in Figure 2, there were no significant between-study variances observed for either gene (Q test *P*>0.24); thus, the fixed-effect model was selected for both genes. Sample size, population region (country), and study age were identified as significant factors that influence the LFC of both genes in case of EA (*P*-value<0.01).

### Potential pathways connecting both genes and EA

**Figure 3:**
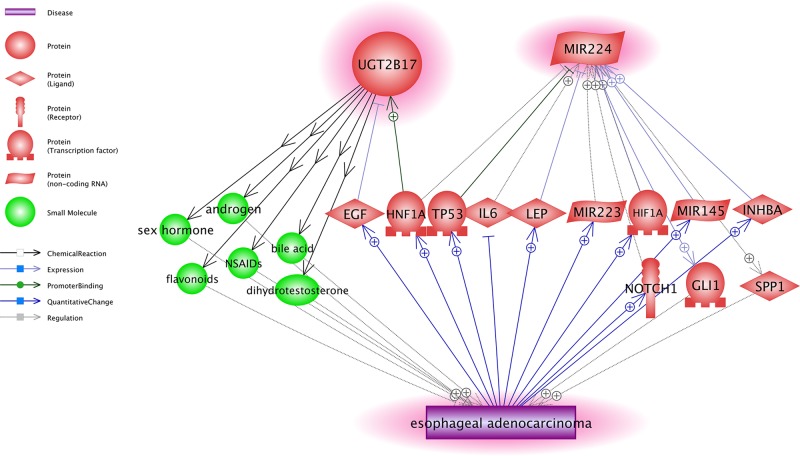
The potential pathways that link UGT2B17 and MIR224 to EA The network was generated using Pathway Studio (www.pathwaystudio.com). Each relation (edge) in the figure has one or more supporting references.

The first step in the procedure approach selected for identification of EA target genes excludes any genes with a known direct relationship to EA, with the requirement of no literature references reporting an association between these genes and EA. To uncover any potential biological relationship between UGT2B17 and MIR224 and EA, Pathway Studio-directed analysis for the functional connections revealed multiple possible pathways that could link these two genes and EA, as shown in Figure 3.

The shortened path analysis was conducted to identify other genes that were linked to both the genes and EA in a directed path. For the details of more pathways were presented in Figure 3, please refer to **EA_ESCC→EA-2Genes_potential pathways**. The reference information includes types of the relationship, the number of underlying supporting reference, and the related sentences where these relationships have been identified and described.

**Figure 4:**
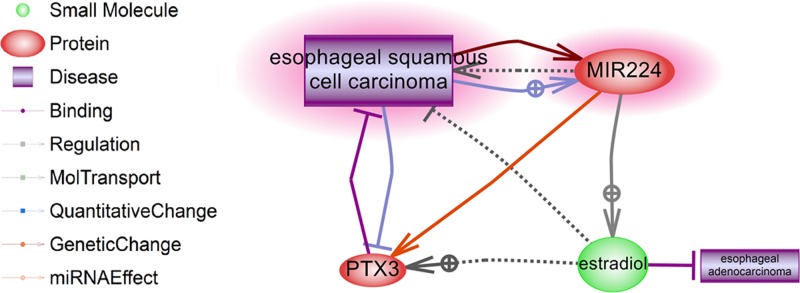
An analysis of the connections of *MIR224* and known contributors to the pathogenesis of ESCC

Additional pathways connecting MIR224 and ESCC are shown in Figure 4. It seems that estradiol (E2) plays a key role in the regulation hub of MIR224 for both EA and ESCC. The reference supporting the relations presented in Figure 4 can be found at **EA_ESCC→Ref for MIR224 regulation hub.**

## Discussion

While the clinical outcomes of two common types of esophageal cancers, ESCC and EA, are comparable, the pathophysiology of these cancers seems to be different. EA arises from the background metaplastic condition of the distal esophagus knowns as Barrett’s esophagus (BE), where, due to long-term gastroesophageal reflux disease, the normal squamous epithelium got replaced by columnar epithelium [18]. The sequence of histopathological changes resulting in ESCC developing esophagitis with atrophic lesions resulting in mild-to-severe dysplasia which leads to appearing of the focal carcinoma *in situ* and finally, invasive cancer [19]. For understanding and optimizing future treatments it is important to understand the specific biology of these cancers. A majority of recent research efforts aimed at showing that EAC and ESCC are different cancer entities [20,21]. Nevertheless, these cancers do share certain commonalities. For example, the alterations in *TP53* locus are the most commonly observed type of genetic change in both EA and ESCC [22]. Other common abnormalities include the changes in the core components of cell cycle machinery encoded by genes *CDKN2A* and *CCND*1 [23]*.*

Our own analysis of commonalities between EA and ESCC showed that the gene sets associated with these two types of esophageal cancer significant overlap (*n*=157, *P*-value =5.14e-143). Furthermore, for 138 out of these 157 genes, a significant enrichment within 102 pathways (*P*-value <1e-26, FDR corrected: q = 0.005) was detected; many of these pathways have been previously implicated in both diseases, such as the ‘aging’ (GO ID: 0007568), ‘regulation of apoptotic process’ (GO ID: 0042981), and ‘regulation of cell proliferation’ (GO ID: 0042127) [24–28]. These results suggest that the pathophysiology of ESCC and EA have at least some genetic pathways in common.

Closer examination of the set of genes associated with ESCC alone (*n*=931) using gene expression mega-analysis showed that expression levels of 28.28% of these genes were also significantly changed in EA as compared with normal esophageal controls (*P*-value <0.05, see in **EA_ESCC→Mega_Analysis**). Moreover, two genes were identified as potential EA biomarkers (*P*-value <1e-10), including UGT2B17 and MIR224. The MLR analysis showed that sample size, population region (country), and study age were significant factors that influence the LFC of both ESCC related genes in case of EA (*P*-value <0.05) (Table 2).

Gene *UGT2B17* encodes for UDP-glucuronosyltransferase 2 (UGT) family enzyme that modifies androgens and xenobiotics by glucuronidation. UGT enzymes detoxify several major tobacco carcinogens, which primarily target the aerodigestive tract. Indeed, tobacco use is a well-known risk factor for both ESCC [29] and EA [30]. Expression of UGT2 enzymes in general, as well as respective penzo[a]pyrene metabolizing activity are detectable in esophageal tissue [31], but expression for a particular UGT2-encoding gene in question, *UGT2B17*, is not reported in esophagus by Human Protein Atlas (link), possibly due to high frequency of UGT2B17 deletion polymorphism in human populations, especially in Asians (66.7%) [32,33].

UGT2B17 is a central enzyme in the steroid-inactivating pathway and is known to modify circulating levels of sex steroids. In particular, testosterone is mainly conjugated by UGT2B17 [34]. Importantly, individuals with intact copies of *UGT2B17* locus have substantially higher testosterone to epitestosterone ratio (T/E) in urine [33]. In this light, it is important to note that EA has marked male dominance with a male-to-female ratio of up to 9:1, pointing that the hormonal balance may play a specific role in the pathogenesis of EA [35,36]. Recent studies show that the cell lines derived from EA respond to stimulation with androgens [37]. In the present study, we show that the levels of mRNA which encodes for UGT2B17 enzyme are up-regulated in EA samples, thus, pointing that the relationship of UGT2B17 and EA certainly warrants further investigation. As the loss of UGT2B17 alleles results in lower testosterone levels, it is tempting to speculate that the expression of UGT2B17 may serve as a key contributor which defines the histological route for the development of esophageal tumors.

Overexpression of miR-224 has been repeatedly detected in esophageal intraepithelial neoplasia and in ESCC samples along with a decrease in the expression of PHLPP1 and PHLPP2 encoding genes which serve as the targets for this oncogenic miRNA 9. Moreover, in ESCC tissues, an increase in the levels of this miRNA is observed at higher TNM stage and pathologic grades. When miR-224 is expressed ectopically in cultured ESCC cells, the proliferation, the migration, and the invasion of these cells increase, while the apoptosis scores lower [9]. In EA, we observed an opposite regulatory trend, with miR-224 messages decreasing in their concentrations in tumors as compared with the controls (Table 2). Importantly, in a previous study of serum exosomes collected from patients with EA, no miR-224 molecules were found in disease-associated exosomes [38].

As expression levels for miR-224 in ESCC and EA trend in the opposite direction, the miR-224 hub should be studied as genetic regulatory networks (GRN) for differentiating regulatory landscapes in EA and ESCC (Figure 4). Notably, miR-224 targets pentraxin 3 (*Ptx3*) [39], which is a potent tumor suppressor for ESCC [40], and stimulates ovarian E2 release [41]. In ESCC, expression of ERβ receptors is driven by E2, and the status of ERβ is closely associated with the unfavorable prognosis, possibly through altering cell proliferation of carcinoma cells [42]. Contrary to ESCC, increased odds of being diagnosed with EA are associated with higher testosterone-to-E2 ratio and other androgen-to-estrogen metrics [43], with estrogen and selective estrogen receptor modulator (SERM) known as suppressors of its growth [44].

Taken together, these observations point to differential response to sex steroids may be the defining feature that differentiates ESCC and EA. It is tempting to speculate that both the increase in expression of UGT2B17 and the lack of miR-224 signaling contribute to strikingly different hormone dependency trends of ESCC and EA.

There were several limitations to this analysis. First, the meta-analysis was performed on the ESCC-specific genes in case of EA using EA expression data. Testing the EA-specific genes in case of ESCC could be a valuable future work. Second, due to the lack of space, the discussions of the present study were focused on the two significant genes selected. However, genes with less significance should be explored, which were provided in the Supplementary Material S1 (**EA_ESCC→Meta_Analysis**) .

In conclusion, our results support the hypothesis that ESCC and EA share some common pathophysiological pathways which are reflected by a significant overlap between the gene sets associated with these two types of esophageal cancer. The genes encoding for UGT2 enzyme UGT2B17 and miR-224 are differentially dysregulated in ESCC and EA tumors. Enhanced expression of UGT2B17 and the lack of miR-224 signaling may contribute to the responsiveness of EA to the male sex steroids.

## Supporting information

**Supplementary Material S1:** 

## Abbreviations

CI: confidence interval
EA: esophageal adenocarcinoma
ESCC: esophageal squamous cell carcinoma
E2: estradiol
GEO: Gene Expression Omnibus
GO: Gene Ontology
LFC: log2 fold change
MLR: multiple linear regression
UGT: UDP-glucuronosyltransferase 2
